# The effects of psychotherapy for depressed or posttraumatic stress disorder women with childhood sexual abuse history: Meta-analysis of randomized controlled trials

**DOI:** 10.1097/MD.0000000000019776

**Published:** 2020-04-24

**Authors:** Jhih-Yuan Lu, Tao-Hsin Tung, Sheng-Ang Shen, Chien Huang, Pei-Shih Chen

**Affiliations:** a Taiwan Joint Commission on Hospital Accreditation, Taipei; b Department of Public Health, Kaohsiung Medical University, Kaohsiung; c Department of Crime Prevention and Correction, Central Police University, Taoyuan; d Department of Medical Research and Education, Cheng Hsin General Hospital, Taipei; e Department of Clinical Psychology, School of Medicine, Fu-Jen Catholic University, New Taipei, Taiwan.

**Keywords:** childhood sexual abuse, depression, meta-analysis, posttraumatic stress disorder, psychotherapy

## Abstract

**Background:**

Depression and posttraumatic stress disorder (PTSD) are the most common mental disorders of women suffered from childhood sexual abuse histories. It has been widely recognized that depression and PTSD may decrease patients’ quality of life. The objective of this study is conducted to explore the effects of psychotherapy for depressed or PTSD women with childhood sexual abuse history.

**Methods:**

We searched the PubMed and Cochrane Library from inception to June 30, 2019. The search strategy is (sexual assault OR sexual crime OR sexual abuse) AND (depression OR PTSD) AND (treatment OR intervention OR psychotherapy) with no restriction on language. Two authors independently selected the studies, assessed the quality of the included studies, and extracted data.

**Results:**

Nine randomized control trials with 761 participants met the inclusion criteria. There were 340 participants in the psychotherapy group and 421 participants in the control group (usual treatment or waiting list). Compared to usual care, improvements were significantly greater in the psychotherapy group. The Beck depression inventory score for depression diagnosis of the psychotherapy group is lower from 4.27 to 8.96 (*P* < .05) than the control group. The client assessment protocols for PTSD, the diagnosis is also lower from 12.4 to 13.71 than the control group (*P* < .05).

**Conclusion:**

The results suggested that psychotherapy is effective in reducing depressed or PTSD women with childhood sexual abuse. Further large-scale high-quality randomized controlled trials with long-term follow-up are warranted for confirming this finding.

## 1 Introduction

Mental disorders are significant public health problems and many studies confirm that have high prevalence globally.^[1]^ Specifically, the duration of untreated depression had a substantial impact on the clinical outcomes of depressed patients. Importantly, a shorter duration of untreated depression is linked with more favorable outcomes in depressed individuals, including depression-related disability.^[2]^ A meta-analysis also indicated that a shorter duration of untreated depression in the 1st episode was related to a higher likelihood of response to antidepressant treatment and remission from depression.^[3]^

Posttraumatic stress disorder (PTSD) and depression both are major mental disorders.^[4–6]^ In addition to serious car accidents,^[7]^ natural disasters,^[8]^ and war,^[9]^ women who experienced of sexual abuse also the main causes of PTSD.^[10,11]^ Some psychotherapy approaches such as supportive psychotherapy, cognitive behavioral therapy, and psychodynamic psychotherapy would provide a potential avenue for intervention to reduce the associated disease burden in subjects with childhood sexual abuse (CSA).^[12,13]^ Furthermore, depression represents big challenges to public health as highly prevalent worldwide. There is increasing evidence that early life experiences and exposures can have long-term effects on health that may manifest as disease later in life.

In females, the highest odds ratios are seen for PTSD (odds ratio = 7.25) in an Australia study.^[14]^ Patient with PTSD and depression may be hard to do normal daily activities, increase fatigue, even feeling worthless or guilty, and thoughts on death or suicide. These symptoms are not a substantive disease, so it is hard to position accurately, based on the above reasons, the difficulty of treatment increase is a serious problem for medical carriers. To improve the symptom of the CSA women who with PTSD and depression, using psychotherapy is the most common treatment. In recent years, there has been a growing interest in and commitment to the combination of psychotherapy and other mental disorder treatment in care settings.^[15,16]^

The aim of this systematic review and meta-analysis was to examine whether the effective of active psychotherapy would produce greater mental health gains than usual care for PTSD and depression women with sexual abuse histories. To the extent that CSA may contribute to these outcomes, some psychotherapy would offer a potential avenue for intervention to reduce the associated disease burden, in addition to the reduction in the burden of disease in women that could be avoided the negative effect on their quality of life.

## 2 Materials and methods

### 2.1 Literature search and search strategy

We searched the PubMed and Cochrane Library from inception to June 30, 2019. The search strategy is (sexual assault OR sexual crime OR sexual abuse) AND (depression OR PTSD) AND (treatment OR intervention OR psychotherapy) with no restriction on language. Two authors independently selected the studies, assessed the quality of the included studies, and extracted data.

### 2.2 Study selection

Studies were included if they met the following inclusion criteria: the study design was a randomized controlled trial, the subjects were depression or PTSD women with CSA history, the experimental group received 4 psychotherapies: cognitive processing therapy, dialectical behavior therapy for PTSD (DBT-PTSD), interpersonal psychotherapy, and structured psychiatric intervention, and the control group received usual care and individual support, and mean difference (MD) and standard deviation were reported in the article. The full texts were checked carefully to see whether there was any potentially related information or not.

### 2.3 Data extraction

Rules for extracting and synthesizing data from selected studies were based PRISMA checklist. The following data were extracted from the included eligible studies through a data extraction form: 1st author, year of publication, country of publication, study period, assigned group, randomly assigned participants, types of any participants, intervention time, and methods used for assessing the severity of depression and PTSD. In addition, we used the Cochrane collaboration tool to assess the risk of bias of the included trials and evaluated the following 7 domains associated with bias of intervention: random sequence generation, allocation concealment, blinding of participants and personnel, blinding of outcome assessment, incomplete outcome data (Attrition bias: it refers to systematic differences between groups in withdrawals from a study lead to incomplete outcome data.

### 2.4 Ethical review

Due to the systematic review and meta-analysis design, the ethical approval was waived and not necessary in this study.

### 2.5 Statistical analysis

The Review Manager 5.3 (The Nordic Cochrane Centre, The Cochrane Collaboration, 2014) was used for meta-analysis. We presented the MD or standardized MD (SMD) with 95% confidence interval (CI) for continuous data. Because the baseline which assesses evidence-based complementary, because the severity of depression and PTSD are different, we used standardized data to adjust the different baseline.

Heterogeneity in meta-analysis refers to the variation in study outcomes between studies. In this study, we used Chi-squared and *I*^2^ inconsistency statistics. *I*^2^ statistic describes the percentage of variation across studies which is due to heterogeneity rather than chance.^[17]^ To the observed other views of this meta-analysis, we used not only the overall but also the outcomes of the Beck depression inventory (BDI), the Clinician-Administered PTSD scale (client assessment protocols, CAPs), and Hamilton depression rating scale (Ham-D), these results used the same measure tools and do not had to adjust the baseline so they presented by MDs. Among of them, BDI had 4 trials, CAPs and Ham-D had 2 trials.

In this study, a *P* value of <.10 indicated significant heterogeneity. *I*^2^ values of 0% to 24.9%, 25% to 49.9%, 50% to 74%, and 75% to 100% were considered as none, low, moderate, and high heterogeneity. A 95% CI for *I*^2^ is constructed using the iterative noncentral Chi-squared distribution method.^[18]^ In addition, we used the fixed-effect model when *I*^2^ was <75% and would have used the random-effects model if *I*^2^ had been 75% or more. For analyzing the continuous data, if the SD was not reported, we estimated SD by SMD and 95% CI.

## 3 Results

### 3.1 Literature search and studies characteristics

**Figure 1:**
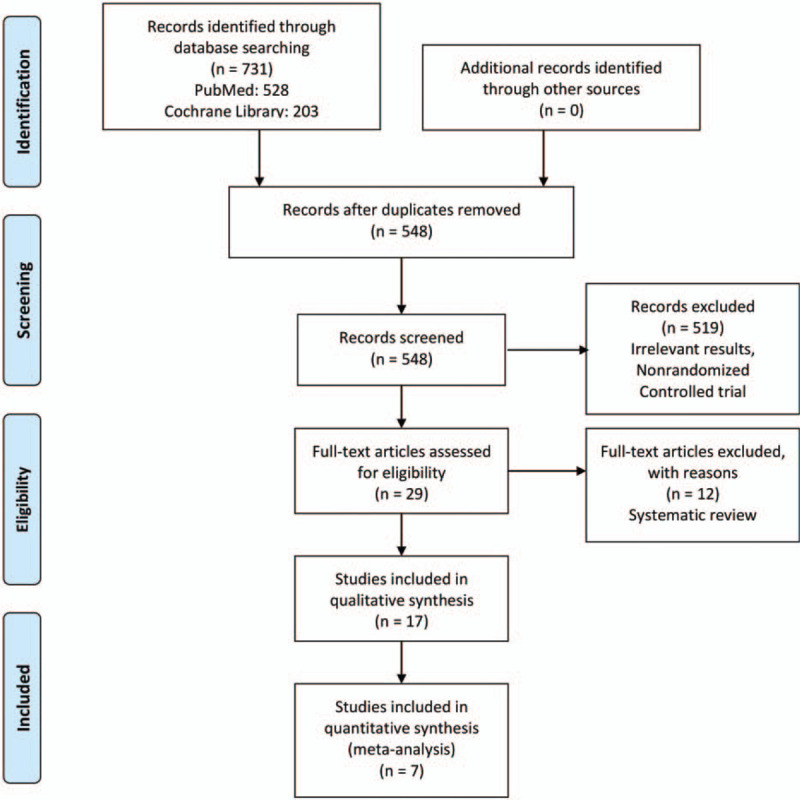
PRISMA (Preferred Reporting Items for Systematic Reviews and Meta-Analyses) flow diagram.

Figure 1 shows that the search process and how to selected studies for systematic review and meta-analysis (PRISMA) guidelines which we followed.^[19]^ We acquired 731 studies from PubMed and Cochrane library, then removed 173 duplicates studies, after excluding nonrandomized controlled trials, systematic review, meta-analysis, and irrelevant results, the rest of it only 7 randomized controlled trials with 761 participants were included in this systematic review and meta-analysis.

**Figure 2:**
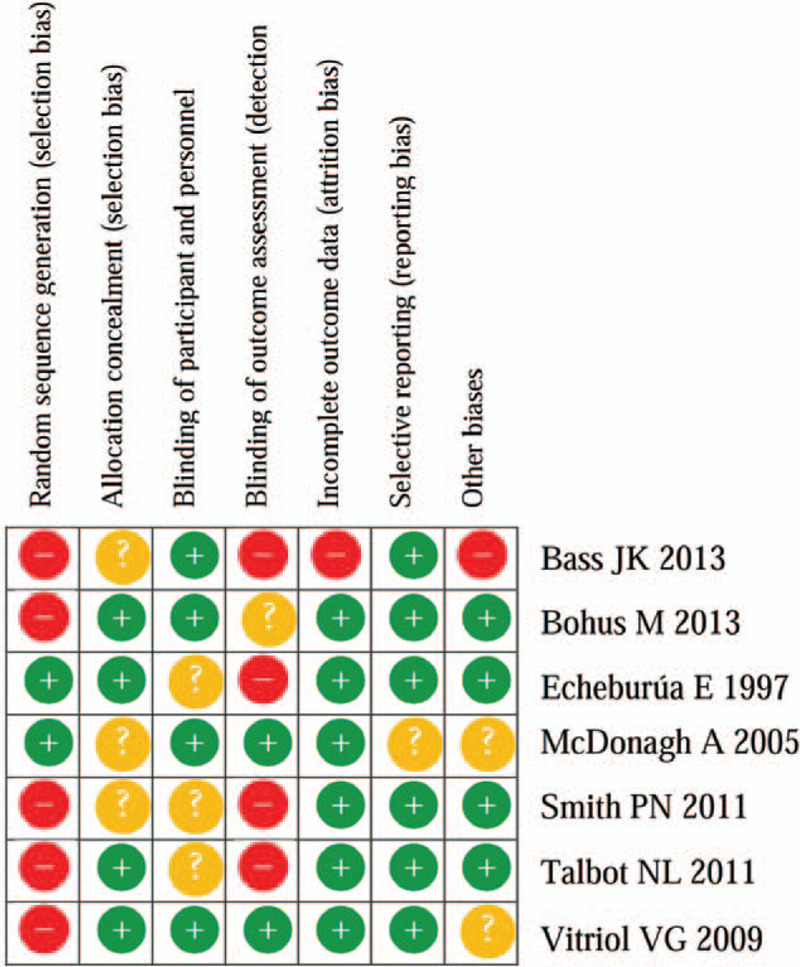
Risk of bias summary: authors’ judgments about each risk of bias item for each included study. CI = confidence interval, SD = standard deviation.

The characteristics of the included trials are summarized in Table 1. These trials are published from 1997 to 2013, The sample size was from 10 to 248, with a total of 761 participants (421 participants in the usual care group and 340 participants in the group of psychotherapy treatment). There are 5 trials had a high risk of random sequence generation because they selected a specific sources of CSA, women with depression or PTSD like villages, limitations of the study include baseline differences in symptom severity between study groups that may limit comparability. Randomization was performed within blocks of two to four villages grouped on the basis of language and proximity, with the assumption that villages close to one another would be similar (Bass et al), department of Psychosomatic Medicine and Psychotherapy at the Central Institute for Mental Health (Bohus et al and Smith et al and Talbot et al) and medical center (Vitriol et al). All 7 trials were not double-blinded because psychotherapists must know what psychotherapy treatment they do (Fig. 2).

**Table 1:**
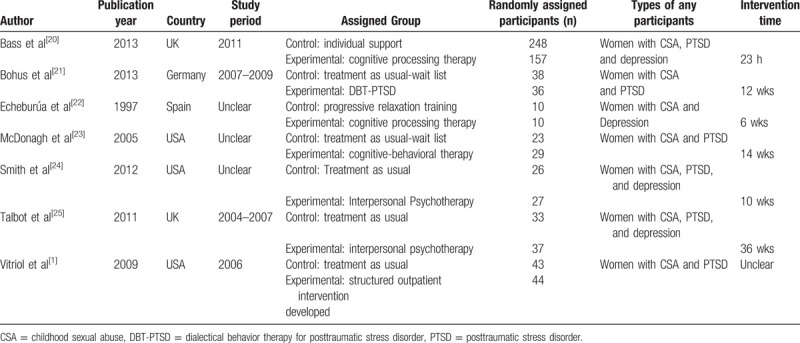
Characteristics of included randomized controlled trials.

It is worth noting that 1 trial had incompleted data (Attrition bias), the originally included participants were 405, but in 6 months after the end of treatment lost some participants, though it was not a large part of them, this study still not presented how many participants lost, that may cause some bias of attrition.^[20]^

### 3.2 Effects of psychotherapy intervention on CSA women with depression and PTSD

**Figure 3:**
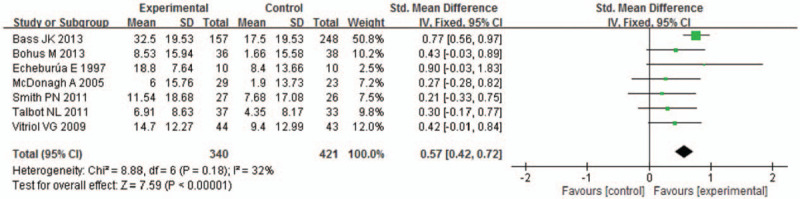
Meta-analysis based on the mean difference between psychotherapy and usual care. CI = confidence interval, SD = standard deviation.

For the overall models, we organized data from the selected trials using fixed models because of low heterogeneity (*χ*^2^ = 8.88, *P* = .18, and *I*^2^ = 32%) (Fig. 3). The pooled SMD was 0.57 (95% CI 0.42–0.72). Then the test for overall effect presented (*Z* = 7.59, *P* < .00001), these data presented the change in score that measured PTSD and depression.

**Figure 4:**
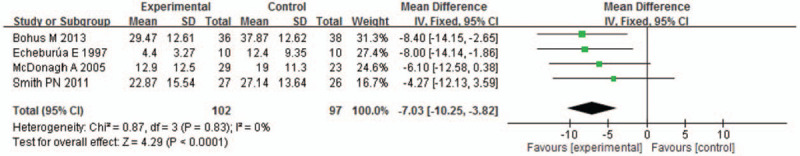
Meta-analysis based on the mean difference between psychotherapy and usual care (BDI). CI = confidence interval, SD = standard deviation.

**Figure 5:**
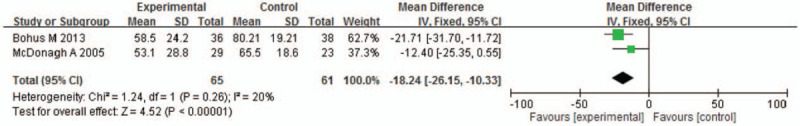
Meta-analysis based on the mean difference between psychotherapy and usual care (Caps). CI = confidence interval, SD = standard deviation.

**Figure 6:**
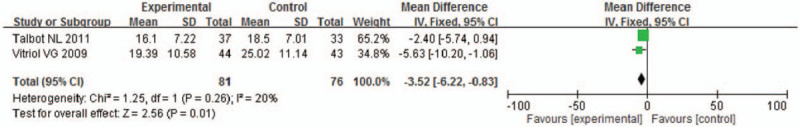
Meta-analysis based on the mean difference between psychotherapy and usual care (Ham-D). CI = confidence interval, SD = standard deviation.

For the BDI models, the pooled MD was −7.03 (95% CI −10.25 to −3.82), test for overall effect presented (*Z* = 4.29, *P* < .0001; *χ*^2^ = 0.87, *P* = .83, and *I*^2^ = 0%) (Fig. 4). For the Caps models, the pooled MD was −18.24 (95% CI −26.15 to −10.33), test for overall effect presented (*Z* = 4.52, *P* < .00001; *χ*^2^ = 1024, *P* = .26, and *I*^2^ = 20%) (Fig. 5). In addition, for the Ham-D models, the pooled MD was −3.52 (95% CI −6.22 to −0.83), test for overall effect presented (*Z* = 2.56, *P* < .01; *χ*^2^ = 1.25, *P* = .26, and *I*^2^ = 20%) (Fig. 6). All the above models were significant differences in the effect of psychotherapy and usual care.

**Figure 7:**
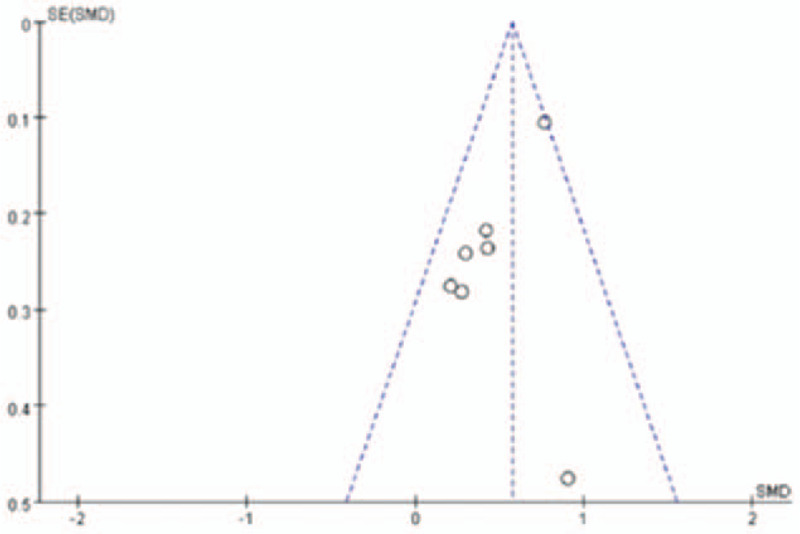
Funnel plot for publication bias estimation.

Funnel plot shows the publication bias of meta-analysis, when the result of the funnel plot is symmetry, indicating no publication bias in this study (Fig. 7). In other words, there is public bias in this study if the funnel plot is asymmetry. According to the basic principle of funnel plot, the power may decrease if the included studies <10 (Golden ratio), and some of these bias are come from the other reporting bias like selecting reporting and regional error. In our meta-analysis, indicating no publication bias and the reason why was funnel plot show symmetry.

## 4 Discussion

### 4.1 Clinical implications

To the best of our knowledge, this study is the 1st systematic review and meta-analysis to examine the effects of psychotherapy on depressed or PTSD women with CSA. Our study results supported psychotherapy is positive effectiveness for CSA women with PTSD and depression. Even if it is very developed now, mental disorders are still having lots of difficulties that we must to overcome and psychotherapy played an important role in it. Along with the increase of CSA women with PTSD and depression, many researchers desire to find an effective treatment to cure these vulnerable groups, after we completed the systematic review and meta-analysis, we gained a positive outcome to improve psychotherapy is really effective for CSA women with PTSD and depression. The results indicate that with appropriate training and supervision, psychotherapeutic treatment can be successfully implemented and can have an effect in settings with few mental health professionals. This therapy holds promise as a community based service for sexual-violence survivors in similar contexts and warrants confirmatory studies and scale-up evaluations.^[20]^

It is important that making a plan for these patients that have severe mental disorder by their past traumatic situations, such as the study we included in our meta-analysis mentioned, the intervention actively screens for patients’ experiences of childhood trauma and focuses on these experiences and patients’ current interpersonal difficulties, with the understanding that current difficulties may be repetitions of past traumatic situations.^[26–28]^ It is also essential to continue to develop, implement, and validate interventions for this patient subgroup that can be delivered in public health settings. The above showed interpersonal psychotherapy, how to overcome depression and PTSD, and so as CBT, DBT-PTSD, and structured psychiatric intervention. Our study improved the importance of psychotherapy for CSA women with depression and PTSD. Despite the advantages for psychotherapy, there was substantial variation in depression outcomes. Multiple factors, independent and converging, may be implicated in suboptimal treatment outcomes. Research is needed to strengthen treatments for women with severe trauma histories and complex psychiatric conditions in community mental health care.^[29]^ And we expected that patients’ relationships with immediate family and intimate partners would be less likely to improve during treatment than relationships with co-workers, friends, or extended family, unless patients received an interpersonally focused intervention that targeted close relationships.^[30]^ It is difficult to develop effective treatment at a realistic level due to not only all of the psychotherapies we included are consuming manpower and money, but also psychotherapist should to be trained for a long time.^[31]^

### 4.2 Clinical practice

The mental health burden of co-occurring major depression and CSA included higher risk of chronic and recurrent depression, comorbid PTSD, borderline personality disorder, suicidal behavior, and heightened shame.^[32–34]^ Evidence-based studies indicated that a sexual abuse history significantly increases the likelihood of adult major depression and that more severe forms of abuse increase 3-fold the risk.^[25]^ The complex clinical scenario observed among adults who have a childhood trauma experience has been viewed as a process of neurobiologic and psychologic vulnerability.^[1]^

This study showed that cognitive behavioral therapy reduced PTST symptoms and combined depressive symptoms. The clearest empirical evidence of effectiveness in treating symptoms related to PTSD found for cognitive behavioral treatment.^[35]^ Although it appears that cognitive behavioral therapy has a clinical positive impact on PTSD symptoms for some female CSA survivors, the relative higher dropout rate implied that a significant proportion of victim women were not willing to complete all courses of this treatment.^[23]^ Therapist differences also could be a factor in the dropout, so that therapist might be treated as a random variable to further draw across therapists in general.

In addition, interpersonal psychotherapy was also suggested in improving psychiatric symptoms and reducing shame among CSA women.^[25]^ Victim women who suffered sexually abused are more likely to experience difficulties maintaining stable intimate relationships, poor communication with intimate partners, and dissatisfied relationship dissatisfaction.^[33]^ They also faced an elevated risk of adult sexual assault, intimate partner violence, and intergenerational transmission of sexual abuse.^[32,36,37]^ Interpersonal psychotherapy reduced the severity of depressive symptoms, PTSD symptoms, feelings of shame, and improved functioning within the family to a greater degree than usual care psychotherapy. Due to the high dropout rates that are typical in community mental health, it is noteworthy that treatment engagement and retention were stronger in interpersonal psychotherapy than in usual care.

### 4.3 Methodologic considerations

In this study, only 7 trials that we included less than the golden ratio of the funnel plot, so the power is the defect that we should to emphasize. A further limitation is that the compositions of usual care is different, it may cause some bias between the experimental group and control group in every trial. Our study is hard to maintain blind because psychotherapist must to know that which psychotherapy they did on patients. Another limitation is about attachment and alliance in the treatment, women who experience CSA tend to report more insecure patterns of attachment and more unstable. Thus, among depressed women with sexual abuse histories, insecure patterns of attachment may interfere with their ability to form a trusting alliance with their therapist and may contribute to the poorer treatment outcomes often seen among patients with insecure attachments.^[38,39]^

## 5 Conclusion

In conclusion, our data suggested that psychotherapy is effective in CSA women with PTSD and depression as an intervention treatment. Both interpersonal psychotherapy and cognitive behavioral therapy were related to sustained symptom reduction. Further large-scale high-quality randomized controlled trials with long-term follow-up are warranted for confirming this finding.

## Acknowledgment

The authors thank the Sunflower Statistical Consulting Company, Kaohsiung, Taiwan for statistical advice.

## Author contributions

**Conceptualization:** Jhih-Yuan Lu, Sheng-Ang Shen, Tau-Hsin Tung

**Data curation:** Jhih-Yuan Lu, Pei-Shih Chen.

**Formal analysis:** Jhih-Yuan Lu, Tau-Hsin Tung.

**Methodology:** Chien Huang, Tau-Hsin Tung.

**Resources:** Sheng-Ang Shen, Tau-Hsin Tung.

**Supervision:** Ruiping Liu.

**Writing – original draft:** Jhih-Yuan Lu, Tau-Hsin Tung.

**Writing – review and editing:** Sheng-An Shen, Chien Huang, Pei-Shih Chen, Tau-Hsin Tung.
